# The adaptation of soldiers to post-service life – the mediating impact of political views on the relationship between violence and adaptation

**DOI:** 10.3389/fpsyg.2023.1131316

**Published:** 2023-08-14

**Authors:** Uzi Ben-Shalom, Abira Reizer, Vincent Connelly, Itamar Rickover

**Affiliations:** ^1^Department of Sociology and Anthropology, Ariel University, Ariel, Israel; ^2^Department of Psychology, Health and Professional Development, Oxford Brookes University, Oxford, United Kingdom; ^3^Department of Psychology, Ariel University, Ariel, Israel

**Keywords:** combat experience, veteran health, political (dis)engagement, adaptation, moral injury

## Abstract

**Introduction:**

The current research explores the association between political views, combat experiences, and the adaptation of soldiers to post-service life. Violent experiences in military service were explored as contributors to both positive and negative dimensions of adaptation, while political views served as possible mediators.

**Methods:**

Three hundred and twenty Israeli veterans participated in the study.

**Results:**

Political views were correlated with adaptation, especially left-to-right voting and anti-militarism. The results support the mediating role of political beliefs (left–right voting and militarism) in the relationship between combat experience and adaptation to post-service life.

**Discussion:**

We contend that political perceptions affect adaptation through sense-making of the combat experiences and the individual processing of these experiences, and the willingness to continue in reserve service, which allows social support and recognition. In addition, they are linked to a sense of bitterness following the reduction of public participation in military and reserve service.

## Introduction

The adaptation of military personnel and veterans to civilian life following military service has been extensively studied, and the link between violence during military operations and the psychosocial effects on those exposed is also well-documented and focused on the negative consequences (Solomon et al., 1992; Shay, 2003; Zerach et al., 2013; Griffin et al., 2019). Direct involvement in killing (Khan et al., 2021), moral injury (Levi-Belz et al., 2022), and physical injury (Xue et al., 2015) have been linked with post-traumatic stress disorder (PTSD) symptoms, a trauma-influenced outlook on life, mental illness, poor physical health, and generally poor adaptation to post-service life.

Researchers have gradually begun acknowledging the positive consequences of exposure to traumatic experiences. Tedeschi and Calhoun (2004) defined Post-traumatic growth (PTG) as “the perceived benefits, positive aspects and the transformation of trauma… that includes positive psychological change, perceived benefits, stress-related growth, flourishing, positive by-products, the discovery of meaning, positive emotions and thriving” (p. 3). This may be when individuals are exposed to a stressful, life-challenging circumstance that can reframe their goals, expand their personal skills and sense of abilities, and cause them to experience personal growth and better appreciate their life (Tedeschi and Calhoun, 2004). Other research expanded their contentions (Teixeira and Pereira, 2013; Wu et al., 2019). Post-traumatic growth (PTG) refers to “positive psychological changes after encountering challenging events” (Wu et al., 2019) (p. 408). Other research expanded their contentions (Teixeira and Pereira, 2013; Wu et al., 2019). Post-traumatic growth (PTG) refers to *“positive psychological changes after encountering challenging events”* (Wu et al., 2019) (p. 408). PTG is not generated by the traumatic event but by reassessing the situation. Indeed, previous work has suggested that PTG occurs among diverse populations, such as those who have been bullied (Ratcliff et al., 2017), firefighters (Ogińska-Bulik and Kobylarczyk, 2016), and survivors of earthquakes and tsunamis (Tang, 2006). There is even an indication that US Army veterans experience PTG (Tsai et al., 2015). A recently published meta-analysis involving 10,181 participants revealed that 52.58% (95%CI = 48.66–56.48%) of people who had experienced traumatic events reported PTG, and this percentage increased among young individuals and those who worked in specific professions (Wu et al., 2019).

Military-to-civilian transitions can be quite difficult. Many Veterans report multiple barriers to a smooth transition and adaptation to civil society (Blais and Renshaw, 2013; Kulesza et al., 2015; Dolan et al., 2022) that lead to a sense of being “stuck” (Shay, 2010). This emphasizes the need to explore the subjective experience of the soldier’s transition process. The adaptation of military soldiers to post-service life has been extensively studied, suggesting that veterans experience mental health disorders and post-traumatic stress, at disproportionate rates compared to their civilian counterparts (Olenick et al., 2015). Most of the empirical work focused on veteran well-being (Caddick and Smith, 2014) and in particular PTSD symptoms among veterans due to its’ increased rate among veternas. Between 2 and 17% of veterans report combat-related PTSD (Richardson et al., 2010), and as many as 11–45% of recently returning veterans meet the diagnostic criteria for PTSD (Helmer et al., 2007; Richardson et al., 2010). Other studies focused on combat veterans’ poor self-related health (Aldwin et al., 2018) or emphasized the need to explore moral injury among veterans (Frankfurt and Frazier, 2016). This narrow clinical focus on pathology tends to result in treating veterans already at the point of crisis, or as “prevention at the edge of the ledge” (Caine, 2019, p. 1) and narrowly focused on one outcome while missing various other adaptation indicsators and did not address to the positive qualities of adaptation to civilian life (Spiro et al., 2016; Wilmoth and London, 2016). Based on the previous literature review we would examine the negative adaptation indicators such as PTSD, physical health, and moral injuries. In addition and based on Spiro et al. (2016) theoretical model we will include potential positive outcomes of the post-military pathways such as life satisfaction (as an indicator of mental health) and military benefits as a sense of pride and military career options. Spiro et al. (2016) suggested that military service may also have later life outcomes both positive (such as social and economic well-being) and negative (such as mortality). However, in the current work we would like to focus on young solders in the transmission to civilian life and we would focus on the short term adaptation aspects as insicated above.

The current study extends this line of research by proposing that we expand our understanding of both the positive and negative consequences of exposure to violence during military service and capture the potential mediators of positive and negative outcomes. Specifically, this study will examine whether political views and anti-militarism may mediate these relationships.

### Political beliefs and veterans’ positive and negative adaptation

Political attitudes and their potential association with veteran adjustment are less well studied than other variables such as age, personality, and gender. Nevertheless, they could also significantly affect this context (Shechner et al., 2007; Ben-Ezra et al., 2015; Molendijk, 2019; Hertz et al., 2022). A reasonable body of research demonstrates that veterans are more likely to be right-leaning and nationalistic in both policy attitudes and voting intentions and more in favor of military-oriented policies than other citizens. This includes those who have served in an all-volunteer military like the US Army (Chatagnier and Klingler, 2016), as well as conscripts from countries such as France (Fize and Louis-Sidois, 2020), Argentina (Ertola Navajas et al., 2022), and Croatia (Lesschaeve, 2020). It has been claimed that this rightward drift is due to those with right-leaning views being more likely to self-select into the armed forces, even in a conscript force (Fize and Louis-Sidois, 2020), as well as the influence of senior NCOs and serving officers, who tend to hold more authoritarian views, strongly endorse patriotism, and thus influence recruits’ political outlook (Chatagnier and Klingler, 2016). The perceived threat to security levels within a national political context may also be substantial in determining veterans’ political attitudes. It has been reported that civilians facing terror attacks demonstrated a shift in their political opinion to the right (Ben-Ezra et al., 2015). Nationalistic politics, especially in a perceived high-threat security context, may also be more prone to highlighting the positive aspects of military service regarding patriotism and service to the country that could reinforce veterans’ pride and sense of competence. This perception of their military efficacy based on their previous service may be related to positive adaptation within a political context where military service is perceived positively and where there is an ongoing security situation (Ben-Shalom et al., 2019). Positive adaptations to life post-service could be indicated by general satisfaction, good physical health, and gleaning positive meaning from one’s previous military service.

Trauma in veterans can also be linked with less political engagement and with more anti-militaristic, left-leaning political views that are less nationalistic (Lesschaeve, 2020). Political beliefs, moral judgments, and trauma are closely linked (Emler, 2003). Recent research has linked these factors with moral injury and resulting detrimental consequences following military operations in a combat zone (Hertz et al., 2022). The effect of political attitudes was also related to moral injury in recent qualitative research (Molendijk, 2019) that asserted that the psychological aspect of trauma is not only internally shaped but interacts with broader societal factors. These factors include ambiguities on the ground resulting from strategic policy and the public’s view of military actions. In turn, these factors reflect on the individual perception and sense-making of military reality (Molendijk et al., 2022). Other research on US conscripts in Vietnam has indicated that exposure to violence and the development of PTSD impacts veterans’ political involvement through lower political efficacy and trust due to feelings of betrayal, isolation, and helplessness, regardless of the social support available (Usry, 2019). The Vietnam veterans’ experience was typified by a disconnect between the veteran and the perception of the military in the politics of the populace and, eventually, the government on their return from duty. This may have predisposed these veterans to have lower political efficacy and trust, and, in fact, the veteran community felt so marginalized that they created their own new social movement to highlight their difficulties with adaptation after military service (Scott, 2017). If the political landscape of a nation is more positively inclined to the military, it would be interesting to see if there are similar negative impacts on veterans’ adaptation to civilian life.

### The Israeli context

Israel, on the surface, retains a critical military system with a citizen-soldier tradition. With constant military and security threats, it has been argued that Israel does not have a clear line between the military and society (Cohen, 2008). Indeed, Israel maintains a large and active reserve component that veterans are constantly entering and exiting and, while doing so, will likely reactivate their military identity (Lomsky-Feder et al., 2008). It is interesting to note that the word “veteran” cannot be translated directly into Hebrew, and the phrase “released from the army” (“*meshurar misherut bzava*”) is instead more appropriate.

There is a tendency in Israel to take military matters for granted, or, in other words, there is a kind of “cognitive militarism” (Kimmerling, 1993). Therefore, adherence to mandatory conscription – or the “People’s Army” – remains a crucial factor in Israel’s political perception of this civic obligation (Ben-Ari et al., 2023). The Israeli military leadership has also maintained the emphasis that the military continues to represent *“very strongly the Israeli idea of ‘Mamlachtiyut’ – a statist national ethos that combines notions of belonging to the same community, due conduct, inclusivity, and engaging for the common good of Israel”* (Peri, 2020, p. 2). This is despite the fact that large parts of the population do not enlist for full military service, and while appearing inclusive, military service in Israel can be highly selective with a pattern of early discharge for those who do not fit what is required (Ben-Ari et al., 2023). This allows the armed forces to benefit from the advantages of both a volunteer system and a citizen army tradition.

Israel has found itself in a protracted military conflict with a constant threat of terror from suicide attacks and cross-border high-trajectory missile fire. Being under constant threat, Israel has often been labeled as a garrison state (Kimmerling, 1993; Ben-Ari, 2018). Commentators and researchers claim that this has affected the Israeli political system. Gradually, the Israeli electorate has tended to move to the right (Peled, 2019). In addition, the debate concerning the occupied territories has never been solved, and hawkish political stances have become more influential. Both processes have increased the prominence of security and the military in driving Israeli politics (Levi and Agmon, 2021). Therefore, it could be that many Israeli veterans – who, like many veterans around the world, are more likely to be politically engaged, more likely to vote, and more likely to be on the right of politics (Usry, 2019) – will maintain their views of the importance of the military in society for continued security purposes. This will be reflected in their political statements and, in Israel, will mirror more comprehensive societal views and may positively impact their adaptation after military service. However, those veterans who hold a left-wing political belief may feel further resentment for being obliged to serve on missions that are against their moral codes. This factor may be related to the development of moral injury. Those who have suffered trauma derived from military service may also be less likely to vote for parties on the right and be more disengaged from politics, which may in turn have a detrimental impact on their adaptation after service. The current paper aims to explore these contentions using the case study of Israeli veterans. The political views surveyed include not only voting intentions but also perceptions of the military institution in Israel. Our study seeks to explore the possibility that the political opinions of veterans will mediate the correlation between experiences of violence in the military with both positive and negative aspects of adaptation to post-service life.

### Hypotheses

Following the literature review, we articulated the following hypotheses:

*H1*: Political views will be associated with adaptation:

*H1a*: Right-wing ideologies will be positively correlated with positive adaptation to post-service life

*H1b*: Belief in the citizen army and militarism will positively correlate with positive adaptation to post-service life.

*H1c*: Anti-militarism will be negatively correlated with positive adaptation indicators

*H2*: Exposure to violence in the military will be negatively related to negative adaptation indicators.

*H3a*: Anti-militarism views will mediate the associations between violence experienced in the military and adaptation to post-service life. Specifically, we expect that exposure to violence during service would be negatively associated with anti-militarism, which in turn would increase negative adaptation and decrease positive adaptation to post service life.

*H3b*: Political beliefs will mediate the associations between violence experienced in the military and adaptation to post-service life. Specifically, we hypothesize that exposure to violence during service would be positively associated with right-wing political voting, which in turn would increase positive adaptation and decrease negative adaptation to post service life.

## Method

### Participants

The participants in this study comprised 411 students from three Israeli universities who had served in the Israel Defense Forces (IDF) and were no longer in regular or compulsory service. The criterion for inclusion in the analysis was service in either combat, combat support, or administrative roles in combat zone areas. In addition, another inclusion criteria for participation in the study was honorable completion of service within the previous 3 years with a maximum age of 30. Specifically, the first 3 years post discharge are critical for shaping the veteran identity and may affect the veteran adaptation to civilian lives. In these critical years the suicidal percentage of veterans are increased; veterans struggle to adjust to civilian life (Ben-Shalom et al., 2023). Ninety-nine respondents were removed from further analysis due to being excluded by these criteria or non-completion of the survey items, so the final sample comprised 312 participants (response rate = 76%). No significant differences were found between the excluded group and the final group in terms of months of service [*t*(409) = 1.1, *p* = 0.25] or distribution of ranks [*χ*^2^(7) = 0.97, *p* = 0.55].

Of the participants, 96% were students or had an academic degree and 93% enlisted after 2011, with an average of 36 months in military service (SD = 23.30). Regarding their rank, 17% completed compulsory service as junior officers (lieutenant to captain), and the rest were junior NCOs – sergeants and staff sergeants – so the participants were broadly representative of a typical IDF reserve cohort (Ben-Shalom and Benbenisty, 2019). The gender distribution was 166 males (53%) and 146 females (47%). Most respondents identified as Jews (*n* = 307, 98%); the rest were Druze, Arabs, or Christians (*n* = 5, 1%). The sample included 89 participants (29%) who perceived themselves as religious, 100 (32%) who identified as traditional, and 113 (36%) who identified as secular; the rest did not disclose their religious orientation.

A large proportion of the participants continued to be assigned to reserve duties (*n* = 194, 62%), while the remainder were not assigned (*n* = 118, 38%). Men were more likely to be assigned to the reserves (*n* = 134, 81%) compared to women (*n* = 60, 41%) [*χ*2(1) = 51.8, *p* < 0.001]. Their activity in the reserves varied: 143 (48%) had not been called up for service in the last 5 years, 89 (30%) had served for at least 1 day, and the rest (*n* = 64, 22%) had served for more than 21 days.

### Procedure

The data was collected using a convenience sample of a self-administered anonymous questionnaire with 138 closed questions. The questionnaires were disseminated through Qualtrics using the social media networks of students from three Israeli universities.

### Measures

#### Background details

The questionnaire included questions on individual details such as gender, political attitudes, religious beliefs, education level, and military background details, including year of enlistment, rank, assignment for reserve duties, role in the military, etc.

#### Adaptation

Adaptation following military service was explored using several indicators that presented positive and negative aspects of adaptation. The positive dimensions related to positive well-being and positive perception of military service, including active participation in the reserves. The negative aspects referred to negative dimensions of psychological well-being. This research comprises seven different indicators, four of which are positive and three are negative.

##### Positive indicators

- *Satisfaction With Life Scale (SWLS)*: Using the SWLS (Diener et al., 1985), the participants assessed five items on a 7-point Likert scale, ranging from 1 (Strongly disagree) to 5 (Strongly agree). An example is the following question: *“In most ways, my life is close to my ideal.”* The internal consistency of the scale was high (Cronbach’s alpha = 0.86).
- *Perceived health*: A single-item question is widely used to assess health (Idler and Benyamini, 1997). Our study used this straightforward strategy, phrasing the question as: *“In general, how would you rate your health status?”* There were five options to answer: Poor, Fair, Good, Very good, and Excellent.
- *Sense of pride*: The participants were told, *“Here are some comments that veterans may sometimes say about their service. Kindly review them and mark your opinion.”* These items included *“I am proud of how I operated in the IDF”* and *“IDF soldiers adhere to the highest moral codes while in action.”* Participants assessed five items on a 5-point Likert scale, ranging from 1 (To a very low extent) to 5 (To a very great extent). The scale’s internal consistency was high (Cronbach’s alpha = 0.80).
- *Military Career Competency Scale*: The participants were told, *“Here are some statements about your feelings concerning your military career.”* The scale comprised six items, including *“I feel that I am capable of performing my military role, should I be required to do so”* and *“I always keep myself updated about new issues related to my military career.”* Participants assessed five items on a 5-point Likert scale, ranging from 1 (To a very low extent) to 5 (To a very great extent). The scale’s internal consistency was high (Cronbach’s alpha = 0.83).

##### Negative indicators

- *The Subjective Traumatic Outlook Questionnaire (STO)*: The STO (Palgi et al., 2017) is a five-item questionnaire with excellent psychometric properties. Participants completed the STO and assessed five items on a 5-point Likert scale, ranging from 1 (Not at all) to 5 (Very much). An example of an item is: *“Looking at your condition, do you feel that you suffer from psychological trauma?”* The internal consistency of the scale was high (Cronbach’s alpha = 0.94).
- *Post-traumatic stress disorder (PTSD) screen*: The four-item PTSD screen was used. This short diagnostic tool proved useful and valid (Hanley et al., 2013). Participants were told, *“Below is a list of reactions that soldiers sometimes experience in response to stressful life experiences. Please mark how much you have been bothered by each problem in the past month.”* The questionnaire used a 5-point Likert scale, ranging from 1 (Not at all) to 5 (Extremely). The scale’s internal consistency was high (Cronbach’s alpha = 0.87).
- *Moral injury*: The Moral Injury Symptom Scale – Military Version – Short Form (Currier et al., 2020) was used. The participants were told, *“Here are some comments that veterans may sometimes say about their service. Kindly review them and mark your opinion.”* The items included *“I am ashamed of myself because of things that I did/saw during my military service”* and *“I feel guilty about things that happened during my military service that cannot be excused.”* Participants assessed eight items on a 5-point Likert scale, ranging from 1 (To a very low extent) to 5 (To a very great extent). The scale’s internal consistency was high (Cronbach’s alpha = 0.89).

#### Political views

Participants’ worldviews were measured by referring to their political voting inclinations and social perceptions of the military.

- *Political voting*: The participants were asked about their political position on a 5-point Likert scale, which covered the options of “Very right-wing,” “Right-wing,” “Center,” Left-wing” and “Very left-wing.” The bulk of the respondents had right-wing political views (*n* = 209, 67%), followed by those with a centrist political view (*n* = 92, 30%). A very small proportion of respondents had a leftist political ideology (*n* = 10, 3%).
- *Social perceptions of the military*: The social perceptions of veterans refer to their attitudes to the military’s role in Israel, especially of the compulsory draft, and their perceptions of militarism and anti-militarism. The scale consisted of 11 items with five response options, ranging from 1 (To a very low extent) to 5 (To a very high extent). Factor analysis with Varimax rotation exhibited a 3-factor solution explaining 55% of the variance (one item was removed for being loaded on two dimensions). The factors comprised:
- *Support for the People’s Army model* – Five items, such as “*I am angry with those who do not serve in the military like myself*,” “*Everyone should serve in the military*,” and “*Those who have not served in the military have not completed their duties to their country*.” The internal consistency of the variable was good (Cronbach’s alpha = 0.79).
- *Anti-militarism* – Three items, such as “*Israel puts too much weight on military-related issues*” and “*Israel does not invest enough resources on civic issues due to unnecessary investments in the military*.” The internal consistency of the variable was fair (Cronbach’s alpha = 0.65).
- *Militaristic view* – Two items: “*Israel must be militarily strong, or it will not survive*” and “*Israel’s security problems can only be resolved using military means*.” The correlation between these variables was medium (Pearson’s *r* = 0.30, *p* < 0.001), and both were merged into a single index called Militaristic View.

#### Combat experiences

The perception of military experiences was measured using a 15-item tool assessing the frequency of violent encounters during military missions. The scale was adapted from the US Combat Experiences Scale (Guyker et al., 2013) but with eight items highly relevant to tensions with terrorists or civilians in current Israeli operations. The scale had four response options: 1 = Never, 2 = Once or twice, 3 = Several times, and 4 = Many times. Factor analysis with Varimax rotation exhibited a 4-factor solution explaining 70% of the variance, and it comprised the following factors:

- *Friction with civilians* – Five items, such as *“I struggled hand to hand with civilians”* and *“I participated in search-and-arrest operations inside houses.”* The internal consistency of the variable was high (Cronbach’s alpha = 0.89).
- *Friction with enemy forces* **–** Four items, such as *“I was exposed to hostile incoming fire”* and *“I fired my weapon at enemy combatants.”* The internal consistency of the variable was good (Cronbach’s alpha = 0.78).
- *Witnessing horrors* – Four items relating to enemy and civilian friction, such as *“I witnessed enemy combatants being seriously wounded or killed”* and *“I witnessed stabbings, gunshots, or hit-and-runs during terror attacks.”* The internal consistency of the variable was good (Cronbach’s alpha = 0.73).
- *Personal injury* – Two items relating to the enemy and civilian friction: *“I was injured in a combat-related incident”* and *“I was wounded while struggling with locals.”* The correlation between the two items was high (r(310) = 0.50, *p* < 0.001).

## Results

The descriptive statistics of the adaptation variables are presented in Table 1.

**Table 1:** Means, standard deviations, and Pearson’s correlations of the adaptation variables.

Variable	*M*	SD	1	2	3	4	5	6	7
1. SWLS	5.03	1.21	1.00	0.320**	0.319**	0.335**	−0.087	0.191	−0.175**
2. PHSI	3.98	0.96		1.00	0.225**	0.141*	−0.223**	−0.163*	−0.194**
3. SOP	4.12	0.74			1.00	0.332**	−0.202**	−0.136*	−0.371**
4. MCCS	3.57	0.91				1.00	0.019	0.100	−0.131*
5. STO	1.49	0.85					1.00	0.530**	0.440**
6. PTSD4	1.51	0.83						1.00	0.330**
7. MI	1.79	0.83							1.00

SWLS, Satisfaction with Life Scale; PHSI,perceived health single item; SOP, sense of pride; MCCS, Military Career Competency Scale; STO, Subjective Traumatic Outlook Questionnaire; PTSD4, 4-item post-traumatic stress disorder screen; MI, moral injury. *p < 0.05, **p < 0.01.

A preliminary analysis of the adaptation variables revealed that the study population is reasonably well-adapted – all the positive variables scored above the middle of the scales, ranging from 3.57 to 4.12 on the 5-point scale and scoring 5.03 on the 7-point scale. In addition, the level of negative adaptation was at the lower end of the scale, and the averages ranged from 1.49 to 1.79 on the 5-point scale.

The intercorrelations reveal support for the pre-assumptions about positive and negative adaptation: The intercorrelations between the positive adaptation variables were low to medium (Pearson’s r ranged between 0.141 to 0.335, *p* < 0.05), while those between the negative adaptation variables were medium to high (Pearson’s r ranged between 0.33 to 0.53, p < 0.001); both groups of variables had negative intercorrelations (Pearson’s r ranged between −0.13 to −0.371, p < 0.05). The correlations between the explanatory and adaptation variables are presented in Table 2.

**Table 2:** Means and standard deviations of the explanatory variables and Pearson’s correlations with adaptation variables.

Variable	*M*	SD	SWLS	PHSI	SOP	MCCS	STO	PTSD4	MI
PV	2.22	0.72	−0.113*	−0.067	−0.299**	−0.007	0.126*	0.144*	0.186**
BPA	3.24	0.86	0.118*	0.083	0.332**	0.249**	−0.031	0.080	−0.081
AM	2.05	0.78	−0.158**	−0.020	−0.271**	−0.223**	0.275**	0.182*	0.340**
MM	3.99	0.68	0.073	0.095	0.338**	0.192**	0.031	0.124	−0.017
FWC	1.83	0.93	0.121*	0.143*	0.151**	0.187**	0.057	0.080	0.044
MF	1.32	0.51	0.066	−0.014	0.102	0.186**	0.231**	0.190**	0.111
WH	1.46	0.57	0.063	−0.026	0.022	0.147**	0.260**	0.334**	0.208**
PI	1.17	45	0.015	−0.074	0.012	0.057	0.262**	0.223*	0.213**

PV, political voting (higher score = left); BPA, belief in People’s Army; AM, anti-militarism; MM, militaristic mindset; FWC, friction with civilians; WFE, friction with enemy forces; WH, witnessing horrors; PI, personal injury; SWLS, Satisfaction with Life Scale; PHSI, Perceived health single item; SOP, sense of pride; MCCS, Military Career Competency Scale; STO, Subjective Trumatic Outlook; PTSD4, 4-item post-traumatic stress disorder screen; MI, moral injury. *p < 0.05, **p < 0.01.

The correlations provided in Table 2 grant preliminary support for the first two research hypotheses: left-wing voting had a low negative correlation with two out of the four positive adaptation variables and a low to medium positive correlation with the negative adaptation variables. At the same time, belief in the People’s Army model positively contributed to three out of the four positive adaptation variables and was unrelated to negative adaptation. Anti-militarism was associated with both positive and negative adaptation. Militaristic views were correlated with only two of the positive adaptation variables.

Indicators of experience of violence in the military were correlated with negative adaptation: three out of the four variables contributed to all three negative adaptation variables. However, the variables were not consistent in their correlation with positive adaptation: only the friction with civilians variable had consistently low correlations with these indicators. In addition, the MCCS was positively correlated with three out of four friction variables.

We computed a series of hierarchical linear regression analyses to better explore the research hypotheses to explain the adaptation variables controlling for gender differences. The three friction variables were merged to avoid the possibility of multicollinearity. The results support the first two hypotheses, although not all of the variables were equally significant. Six out of seven adaptation variables were significantly explained to various degrees (R2 indicators ranged between 0.059 to 0.262, *p* < 0.01). Political voting and anti-militarism were negatively related to both positive and negative adaptation indicators. Belief in the People’s Army and militaristic mindset were positively associated with positive adaptation indicators but were non-related not to negative adaptation indicators. In addition, women present more negative adaptation compared to men. Friction was negatively related to one positive adaptation indicator (MCCS). The results are shown in Table 3.

**Table 3:** Hierarchical regression analysis for predicting positive and negative adaptation.

		SWLS		PHSI		SOP		MCCS		STO		PTSD4		MI	
		*β*	*t*	*β*	*t*	*β*	*t*	*β*	*t*	*β*	*t*	*β*	*t*	*β*	*t*
Step 1															
Gender	−0.04	−0.7	−1.45	−2.57**	−0.094	−1.65	−0.00	−019	0.13	2.42**	0.11	1.76	0.12	2.20**	*R* ^2^	0.02		0.01**		0.00		−0.00		0.01**		0.00		0.01		*F*(1,308)	0.50		6.16**		2.73		0.03		5.86**		3.11*		9.32**	
	Step 2															
	Gender	−0.03	−0.56	−0.17	−2.88**	−1.40	−2.72**	−0.02	−0.40	0.23	4.28**	0.21	3.36**	0.20	3.72***	PV	−0.73	−1.22	−0.04	−0.68	−1.87	−3.59**	0.08	1.52	0.06	1.12	0.08	1.22	0.10	1.93	BPA	0.90	1.46	0.07	1.25	0.23	4.40**	0.17	2.92**	−0.05	−0.87	0.05	0.80	−0.07	−1.34	AM	−1.44	−2.45*	−0.01	−0.22	−0.20	−3.59**	−0.23	−4.16***	0.26	4.76***	0.22	3.40***	0.32	5.81***	MM	0.12	0.19	0.073	1.16	0.20	3.77**	0.13	2.27**	0.06	1.02	−0.09	0.92	0.03	0.622	CSF	0.12	2.15*	−0.64	−1.09	−0.00	−0.03	0.15	2.75**	0.24	4.45***	0.29	4.57**	0.13	2.40**	∆*R*^2^	0.057		0.008		0.256		0.127		0.14		0.15		0.15		*R* ^2^	059**		0.02		0.262***		0.128**		0.15***		0.16***		0.16***		*F*(5,303)	3.19**		2.31*		19.29**		8.53**		10.13**		8.08**		9.24**	

Gender: 0 = male, 1 = female; PV, political voting (higher score = left); BPA, belief in People’s Army; AM, anti-militarism; MM, militaristic mindset; CSFT, Combined friction scale; SWLS, Satisfaction with Life Scale; PHSI, Perceived health single item; SOP, sense of pride; MCCS, Military Career Competency Scale; STO, Subjective Traumatic Outlook; PTSD4, 4-item post-traumatic stress disorder screen; MI, moral injury. *p < 0.05; **p < 0.01; ***p < 0.001.

### Hypothesis 3 model testing

To test our third hypothesis, two confirmatory factor analyses (CFA) using SEM-AMOS were performed before testing the hypothesized model. The first CFA consisted of five variables: combat experiences, positive adaptation indicators, negative adaptation indicators, anti-militarism, and political voting. The combat experience latent factor comprised four parceling measures (friction with civilians, friction with enemy forces, witnessing horrors, and personal injury). The positive adaptive latent factor included four parceling measures (SWLS, perceived health, sense of pride, and MCCS). The negative adaptive latent factor comprised three parceling measures (STO, 4-item PTSD screen, and moral injury). Parceling creates several measures for the latent construct, reducing the measurement error and the risk of spurious correlations (Little et al., 2022). Items were assigned to parcels using Little and colleagues’ recommendations (2002). The measurement model showed an acceptable fit with the data: *χ*^2^(35) = 155.691, *p* = 0.00, *χ*^2^/df = 2.78, CFI = 0.900, TLI = 0.849, RMSEA = 0.006. The single-factor measurement model for the scale showed a poor fit with the data: *χ*^2^(26) = 529.166, p = 0.00, *χ*^2^/df = 8.141, CFI = 0.492, TLI = 0.390, RMSEA = 0.152.

**Figure 1:**
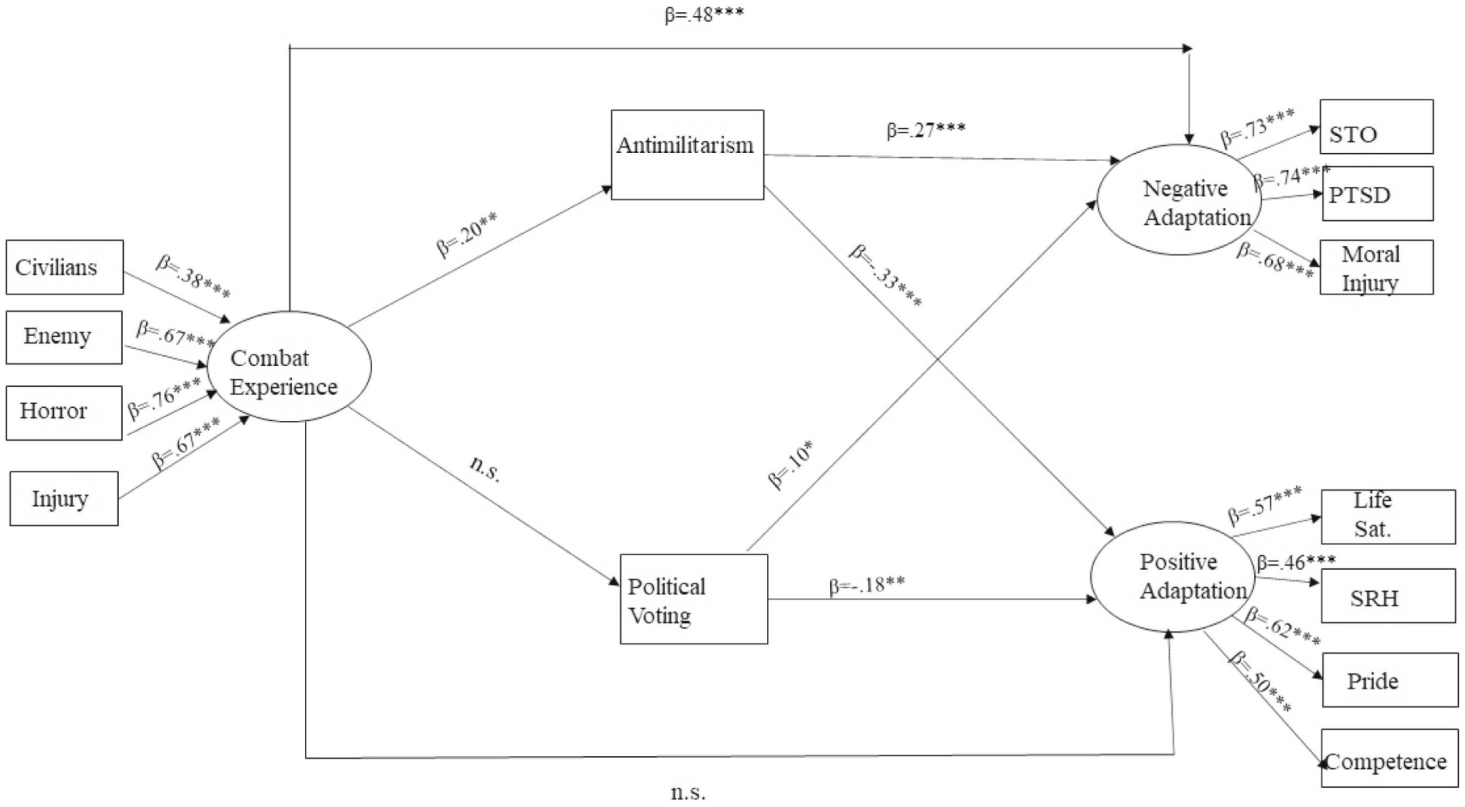
Mediation effects of anti-militarism and political voting in the associations between combat experience and veteran adaptation outcomes, controlled by gender and being an active reservist. *p < 0.05; **p < 0.01; ***p < 0.001.

Structural equation modeling (Arbuckle, 2010) examined the hypothesized theoretical model. The model included direct paths between combat experiences and positive and negative adaptation and indirect paths through political voting and anti-militarism. We also controlled for gender and active reserve service, as both measurements were associated with positive and negative adaptation. The results of the hypothesized model showed an acceptable model fit: (*χ*^2^(42) = 224.754; *χ*^2^/df = 2.881, *p* < 0.001; CFI = 0.875; TLI = 0.831; NFI = 0.824; IFI = 0.878; RMSEA = 0.007). The model is presented in Figure 1. Non-significant associations were revealed in the theoretical model between combat experiences and political voting (*β* = 0.051, *p* = 0.421) and between combat experiences and positive adaptation (*β* = 0.030, *p* = 0.693). Specifically, the non-significant associations between combat experiences and positive adaptation imply that the direct associations between combat experience and positive adaptation outcomes are fully mediated by anti-militarism. Therefore, the full mediation model of combat experiences and positive adaptation is preferred, as it presents a more parsimonious result. The results of the comparison model provided an acceptable model fit and are very similar to those of the hypothesized model and did not improve the model fit; therefore, we adopted the hypothesized model.

As presented in Figure 1, there is a positive association between combat experience and anti-militarism (*β* = 0.200, *p* = 0.002), a positive association between anti-militarism and negative adaptation (*β* = 0.273, p < 0.001), and a negative association between anti-militarism and positive adaptation (*β* = −0.333, p < 0.001). In addition, left-wing political voting is negatively related to positive adaptation (*β* = −0.185, *p* = 0.007) and positively associated with negative adaptation (*β* = 0.100, *p* = 0.044). Finally, there is a direct association between combat experiences and negative adaptation (*β* = 0.484, p < 0.001). This implies that the association is partly mediated by anti-militarism.

The bootstrapping technique using the confidence-interval method was applied to examine the mediation hypotheses. The results indicated that anti-militarism mediated the associations between combat experience and negative adaptation, suggesting that increased levels of combat experiences can increase anti-militarism, which ultimately increases the veteran’s negative adaptation (indirect effect = 0.060, p < 0.001, 95% CI = [0.023, 0.155]). The results further indicated that anti-militarism mediated the associations between combat experience and positive adaptation, suggesting that increased levels of anti-militarism can explain the *negative* association between combat experience and positive adaptation (indirect effect = −0.076, *p* = 0.001, 95% CI = [−0.155, −0.025]). Thus H3a was supported. However, the association between combat experiences and political voting was non-significant, as well as the mediating role of the political vote in the associations between combat experiences and positive or negative adaptation indicators. Therefore, H3b was not supported.

## Discussion

This paper explored social factors that intervene in the relationship between experiences of violence in the military and both positive and negative aspects of the adaptation of veterans to post-service life. In addition, the study mapped the social mechanisms that explain these associations.

The findings indicated that exposure to military violence is associated with negative consequences, such as PTSD, trauma, and moral injury. The results, therefore, support H2, although exposure to violence was also associated with a single positive indicator of military competence. This finding could be interpreted as a result of better military training related to a higher sense of military competency. While most other studies have focused on the negative consequences of exposure to military violence, the current study expands previous research by suggesting that veterans may reassess their exposure to violent experiences and feel proud and competent, with increased perceptions of life satisfaction and health. Interestingly, military friction affected one positive adaptation indicator of perceived military competence. Probably because soldiers who experienced a high amount of combat were deployed with better combat units, had better training, and had a higher perception of their military prowess.

Our sample consisted of veterans in Israel who have been through compulsory service and are, on the whole, well-adapted to civilian life. They have relatively low levels of negative adaptation and high levels of positive adaptation. This reflects the results found among veterans in many countries and may also suggest that military service can create an experience of resilience. According to Bonanno (2008), in the face of challenging and sometimes traumatic circumstances, one needs to cope with ongoing stressors and adopt more skills, abilities, and knowledge to manage psychological challenges more effectively (Chen and Bonanno, 2020). Even though most systematic reviews of events after traumatic experiences tend to be biased toward the negative consequences (Galatzer-Levy et al., 2018), previous studies suggest that an average of around two-thirds experience resilience and PTG (Bonanno, 2008).

### The political context of veterans’ adaptation

We suggest that the political context can account for the unique associations between exposure to military violence and positive–negative outcomes of adaptation. Our results reveal that political views are relevant to the post-service adaptation of veterans in general and perhaps in the Israeli context in particular. Our contention is partially based on the mobilization principal of professional militaries in which enlistment is voluntary. Thus individuals with anti-military beliefs are less likely to volunteer for service. Contrary, Israel relies on compulsory mandatory service, including individuals with anti-military beliefs. We also found that combat exposure is associated with higher levels of negative adaptation, as has been found in much research on veterans. However, in Israel, this is mediated through high levels of antimilitarism, as has been reported with regard to other nations’ veterans (Molendijk, 2019; Lesschaeve, 2020). Lower levels of antimilitarism, in contrast, mediate the associations between violent combat experiences and more positive indicators of adaptations.

We did not find evidence to support the notion that political voting mediates the association between exposure to violence and adaptation. This could be because the left–right spectrum of political views may not necessarily imply a complete rejection or alienation from all military operations. Antimilitarism, on the other hand, which we have identified as a relevant factor in our study, refers to a more profound opposition to the use of military force in general. Therefore, it is possible that antimilitarism is a more critical factor in the relationship between exposure to violence and adaptation than political voting. Another explanation of the lack of significant association between exposure to violent combat experiences and political beliefs related to the stable aspect of political beliefs. There is an accumulative knowledge in psychology, political science, and sociology literature arguing that personality and demographic are solid predictors of political ideology, political beliefs and political behaviors and voting (Blais and St-Vincent, 2011; Furnham and Cheng, 2019). For example, HEXACO personality traits were associated with right wing views and those personality traits have a long term influence party identification over time (Leone et al., 2012). There is also a significant relationship between genes, personality, and politics. Political views can be shaped during childhood (Verhulst et al., 2012; Mayer et al., 2020).

Militarism in Israeli society is often contended to be a cultural phenomenon deeply rooted in the culture of Israel since its early days (Ben-Eliezer, 1995; Ben-Ari, 2018; Shklyan, 2022). Militaristically minded Israelis are thus programmed to view diplomatic, strategic, and social issues through a military, warlike lens. This notion has persisted, although the nature of Israeli wars has changed, and the level of existential threat to the existence of Israel has dramatically declined (Ben-Eliezer, 2012). Israeli schools are sometimes presented as essential tools for inculcation of this (Lomsky-Feder, 2011; Sherzer, 2021). Alignment with these more expansive societal views of the importance of the military can mitigate the negative impact of combat experiences. It is important to remember that positive adaptation, in this case, also includes pride in one’s own role in the military and high levels of ongoing military competence. Given many combat veterans are liable for active reserve duty in Israel, this is not necessarily a theoretical set of beliefs and could be based on reserve service experience as much as on previous full-time service.

Political beliefs, moral judgment, and trauma are closely related (Emler, 2003). Recent research has linked these factors with moral injury and resulting detrimental consequences following military operations in a combat zone (Hertz et al., 2022). Combat experiences can involve actions (or inactions) that violate personal moral and ethical codes. People try to extract meaning from such difficult positions to deal with them, which can result in the rejection of the institution that puts the individual in that difficult moral and ethical position. This is especially the case if military commanders were perceived to have put political considerations or the reputation of the army institution before their troops’ well-being and safety (Molendijk, 2019). A sense of betrayal by both the military and political leadership can result from the experience of being in a “counterfeit universe” (Lifton, 1992), as Vietnam veterans labeled it, where the personal experience of violence in combat and the personal sense of mission failure is at odds with the prevailing military and political narrative (Smith and Freyd, 2014).

Israeli society, whose armed forces’ conscripts are selectively drawn, has a positive stance toward the military and its role in security. Political parties in power are increasingly supportive of security-based solutions to perceived threats. Anti-militarism is thus at odds with much of the political discourse in Israel regarding the military that veterans are exposed to. Positive veteran adaptation is linked to drawing positive meaning from military service. It is no surprise that attempting to make meaning of combat experience through the rejection of the army institution is difficult to do. At the same time, society extols the value of the military, so it is more challenging to heal mental and moral injuries. Thus, the sense of threat to personal matters, reflected in the anti-military stance, is one possible source of moral damage manifested in negative adaptation.

Our results imply that the individual adaptation perspective correlates with political views. How do they coincide? The moral stance of an individual may be influenced by their social construction of reality. Acceptance of criticism and social debate about the military, could see this reality dynamically change, and the individual thus reacts to a (de)construction of the morality of their actions. At the same time, for other service members, their political views motivate different perceptions and justifications for using force.

### The concept of adaptation

Unlike the majority of veterans in professional militaries, the bulk of veterans in the sample in this study are active reservists. As such, our assumption was that adaptation must be measured not only as a negative aspect of psychological adaptation but also as a positive aspect of wellbeing and the perceived high functioning of the military. We do not assume that our participants are traumatized and that our results indicate a form of PTG. In our study, we explore the adaptation indicators, which can be good or bad, and relate them to social perceptions of social reality. The results indicate that this approach is valuable and valid. Both dimensions of adaptation were separated from each other and were explained by different sets of predictors. In addition, active reservists were highly adapted to their post-service context, although some results indicate that they faced challenges following the violence they experienced in the military. In the Israeli culture, there is a notion of “shooting and crying,” meaning that even when soldiers are reluctant to engage in violence, they should still participate in military missions. At the same time, holding anti-military views or politically left-wing beliefs takes a toll on this complicated amalgamation of ideas. Being called up to reserve service again can result in both pride and pain.

The results point to three social forces that impact adaptation: political views, perception of militarism, and anti-militarism. How do they manifest themselves? On the one hand, there is the self-selection of recruits who are prone to right-wing political views (Fize and Louis-Sidois, 2020), which, in turn, strengthens right-wing political opinions in the military. Such a view was recently studied in the Israeli context (Ben-Ari et al., 2023). Another intriguing consideration is the organizational factor of ongoing reserve service. Being organized in a reserve unit may contribute to a veteran’s positive self-perception and higher efficacy, as well as provide social support and expectations for good conduct (Gazit et al., 2021).

### Limitations and future research

The current research has revealed the association of political views on the relationship between combat experiences and the post-service adaptation of veterans. This study, however, was conducted among students in a unique military system using a cross-sectional design and a convenient sample. Many of the participants are reservists and hence are possibly different than veterans. To enhance the study’s external validity, a larger sample with a cross-national comparison and longitudinal design is needed. Such future research should also be conducted among reservists and veterans to better explore the meaning of being a reservist or veteran and how this is interlinked with both negative and positive aspects of well-being.

In the current study women combatants were lower in physical health and experienced negative adaptation indicators with higher levels of moral injuries and subjective perception of traumatic experience compared to men. These findings are in line with previous reviews indicating that female veterans are more vulnerable to psychological and physical health indicators and have lower levels of psychological resilience compared to male veterans (Neilson et al., 2020; Adams et al., 2021).

In addition, our results should further enable the development of relevant interventions for soldiers and veterans who have experienced continuous exposure to trauma. Finally, focusing on the positive psychological changes of veterans would reinforce certain aspects of military experiences, reminding scholars and military practitioners that stressful events can also bring about some positive changes (Meyerson et al., 2011).

## Data availability statement

The raw data supporting the conclusions of this article will be made available by the authors, upon reasonable request.

## Ethics statement

The studies involving human participants were reviewed and approved by the IRB of Ariel University (number: AU-SOC-UBS-20220214). The patients/participants provided their written informed consent to participate in this study.

## Author contributions

UB-S: conception and design of the study and wrote the first draft of the manuscript. AR: performed the statistical analysis and wrote sections of the manuscript. VC: wrote sections of the manuscript. IR: collection of data. All authors contributed to manuscript revision, read, and approved the submitted version.

## Conflict of interest

The authors declare that the research was conducted in the absence of any commercial or financial relationships that could be construed as a potential conflict of interest.

## Publisher’s note

All claims expressed in this article are solely those of the authors and do not necessarily represent those of their affiliated organizations, or those of the publisher, the editors and the reviewers. Any product that may be evaluated in this article, or claim that may be made by its manufacturer, is not guaranteed or endorsed by the publisher.
